# Audit: children & young peoples's services depression pathway Tees, Esk & Wear Valley Trust wide compliance

**DOI:** 10.1192/bjo.2021.894

**Published:** 2021-06-18

**Authors:** Mary Parker, Veenu Gupta

**Affiliations:** Tees, Esk and Wear Valleys NHS Foundation Trust

## Abstract

**Background:**

This complete cycle consists of an audit assessing compliance with the evidence based person-centred pathway of care for Depression in Children and Young People across the Trust, completion of an action plan and re-audit of progress, aiming to improve patient care. The pathway was derived from NICE Quality Standard 48/Clinical Guideline 28, updated for NICE Guideline 134 (2019) and includes comprehensive assessment considering comorbidities, social, educational and family context, parent/carer Mental Health assessment and plan for treatment including psychological therapies as first line treatment in mild depression.

**Method:**

The Audit tool was compiled from the above evidence based pathway and NICE guidance. Each of the 26 community teams were requested to select 5 cases on the pathway who had completed a minimum of 6 treatment sessions (final sample size n = 61). The results were analysed for compliance against the pathway and compared with previous results by the clinical audit team.

**Result:**

The results showed areas of good practice, maintained and improved on re-audit, with over 90% compliance in key evidence based areas regarding consideration of comorbidity, social and educational context and 100% compliance in offering psychological interventions.

Improvement was obtained in some areas highlighted in the previous audit e.g. poor recording of ICD 10 diagnosis in medical records, 19%, improved to 30%, and less than 40% recording of symptom tracking via the RCADS (Revised Children's Anxiety and Depression Scale) monitoring improved to over 50%. There had been a failure to record identification or referral to other pathways/services for mild depression in the 16-18 age group with 0% compliance; this improved to 82% and 100% respectively.

Areas still needing improvement were highlighted including recording of weekly monitoring of medication side-effects for first 4 weeks (43%) and a referral of parent/carers to mental health services after identifying issues (40%).

Response to the audit also improved significantly from 29% of teams not responding in the initial audit to a limitation of only 1 of 26 (4%) at re-audit.

**Conclusion:**

This audit cycle has demonstrated that use of an evidence based approach has been instrumental in improving patient care. The Audit evidenced areas of good practice in holistic assessment and use of psychological therapies and importantly highlighted areas of significant improvement needed including initial monitoring of medication response and referral onwards of parents/carers with mental health issues. Continuous improvement in patient care is planned via a targeted action plan, and further re-audit.

